# Conditionally mutant animal model for investigating the invasive trophoblast cell lineage

**DOI:** 10.1242/dev.202239

**Published:** 2024-01-15

**Authors:** Khursheed Iqbal, Esteban M. Dominguez, Brandon Nixon, Ayelen Moreno-Irusta, Benjamin Crnkovich, Regan L. Scott, Ha T. H. Vu, Geetu Tuteja, Jay L. Vivian, Michael J. Soares

**Affiliations:** ^1^ Institute for Reproductive and Developmental Sciences, University of Kansas Medical Center, Kansas City, KS 66160, USA; ^2^ Department of Pathology and Laboratory Medicine, University of Kansas Medical Center, Kansas City, KS 66160, USA; ^3^ Department of Genetics, Development, and Cell Biology, Iowa State University, Ames, IA 50011, USA; ^4^ Bioinformatics and Computational Biology Interdepartmental Graduate Program, Iowa State University, Ames, IA 50011, USA; ^5^ Division of Clinical Genetics, Department of Pediatrics, Children's Mercy Research Institute, Children's Mercy Kansas City, Kansas City, MO 64018, USA; ^6^ Department of Obstetrics and Gynecology, University of Kansas Medical Center, Kansas City, KS 66160, USA; ^7^ Center for Perinatal Research, Children's Mercy Research Institute, Children's Mercy Kansas City, Kansas City, MO 64108, USA

**Keywords:** Placenta, Invasive trophoblast cells, Uterine–placental interface, PRL7B1, Cre recombinase, Rat

## Abstract

Placental development involves coordinated expansion and differentiation of trophoblast cell lineages possessing specialized functions. Among the differentiated trophoblast cell lineages are invasive trophoblast cells, which exit the placenta and invade the uterus, where they restructure the uterine parenchyma and facilitate remodeling of uterine spiral arteries. The rat exhibits deep intrauterine trophoblast cell invasion, a feature shared with human placentation, and is also amenable to gene manipulation using genome-editing techniques. In this investigation, we generated a conditional rat model targeting the invasive trophoblast cell lineage. Prolactin family 7, subfamily b, member 1 (*Prl7b1*) is uniquely and abundantly expressed in the rat invasive trophoblast cell lineage. Disruption of *Prl7b1* did not adversely affect placental development. We demonstrated that the *Prl7b1* locus could be effectively used to drive the expression of Cre recombinase in invasive trophoblast cells. Our rat model represents a new tool for investigating candidate genes contributing to the regulation of invasive trophoblast cells and their roles in trophoblast-guided uterine spiral artery remodeling.

## Teaser

**Summary:** A conditional rat model targeting the invasive trophoblast cell lineage has been generated that would be helpful in studying genes that play an important role during placental development.

## INTRODUCTION

The placenta creates the environment in which the fetus develops (Maltepe and Fisher, 2015; Burton et al., 2016). Two main functions are ascribed to the placenta and trophoblast cells, its main cellular constituents: (1) modification of the maternal environment to support pregnancy and (2) a role as a barrier regulating nutrient/waste flow to and from the fetus (Soares et al., 2018; Knöfler et al., 2019). Trophoblast cells are specialized to achieve these important tasks. Hemochorial placentas, as seen in humans and some other species including mouse and rat, possess trophoblast cell specializations that facilitate their entry into the uterine parenchyma and restructuring of uterine vasculature, which creates a direct interface between maternal blood and the trophoblast cell barrier (Pijnenborg et al., 2006; Pijnenborg and Vercruysse, 2010; Soares et al., 2018). The intrauterine migratory abilities of these cells are better developed in the human and rat than in the mouse, which exhibits shallow invasion (Ain et al., 2003; Pijnenborg and Vercruysse, 2010; Soares et al., 2012; Shukla and Soares, 2022). In human, these cells are referred to as extravillous trophoblast (EVT) cells, whereas the generic term, invasive trophoblast cells, is used to describe this cell population in the rat. Regulatory events controlling development of EVT and invasive trophoblast cell populations exhibit elements of conservation and are beginning to emerge from experimentation with human trophoblast stem cells and genetically modified rat models (Chakraborty et al., 2016; Muto et al., 2021; Varberg et al., 2021; Kozai et al., 2023; Kuna et al., 2023; Vu et al., 2023).

It is evident that some regulatory pathways controlling invasive trophoblast cells also contribute to earlier phases of placentation or more broadly to embryogenesis (Hemberger et al., 2020; Scott et al., 2022; Vu et al., 2023). Lentiviral strategies targeted to trophectoderm of the blastocyst have been developed to manipulate gene expression in mouse and rat trophoblast cell lineages and address some of these issues (Georgiades et al., 2007; Okada et al., 2007; Lee et al., 2009). Conditional mutagenesis has also become a mainstay for mouse placental research (Woods et al., 2018). Several trophoblast cell-specific regulatory sequences have been used to direct Cre recombinase to an assortment of different mouse trophoblast cell lineages (Wenzel and Leone, 2007; Hu and Cross, 2011; Mould et al., 2012; Ouseph et al., 2012; Zhou et al., 2012; Crish et al., 2013; Pimeisl et al., 2013; Nadeau and Charron, 2014; Outhwaite et al., 2015; Kong et al., 2018; Wattez et al., 2019; Ozguldez et al., 2020). Research in rat has lagged, and model systems for the generation of conditional mutations within the rat trophoblast cell lineage are yet to be reported.

In this research project, we utilized the prolactin family 7, subfamily b, member 1 (*Prl7b1*) locus as a host for Cre recombinase. *Prl7b1* expression within the placentation site is mapped, the consequences of a *Prl7b1* null mutation described, and invasive trophoblast cell-specific actions of a *Prl7b1-Cre* recombinase rat model defined.

## RESULTS

**Fig. 1.**
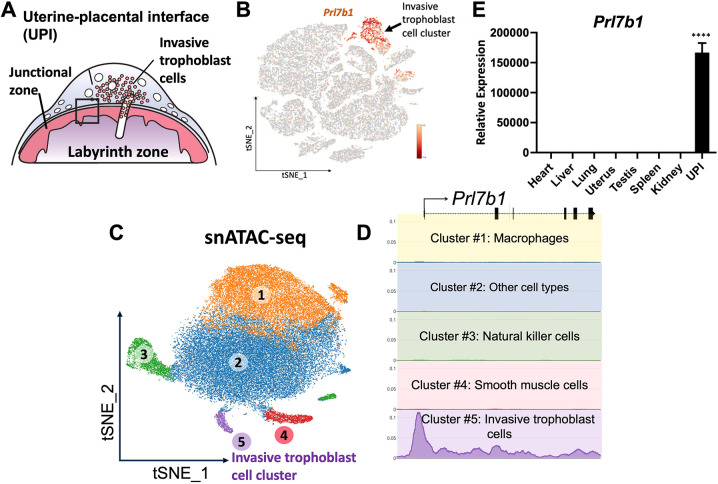
***Prl7b1* expression is specific to invasive trophoblast cells.** (A) Schematic depicting the late gestation rat placentation site. Invaded trophoblast cells are present in the UPI. (B) tSNE (t-distributed stochastic neighbor embedding) plot showing cell clustering on gd 19.5 UPI tissue samples, including the invasive trophoblast cell cluster (re-analyzed from Scott et al., 2022). (C) t-SNE projection of single nuclei isolated from gd 19.5 UPI tissue samples and processed for snATAC-seq (re-analyzed from Vu et al., 2023). (D) Chromatin accessibility profile of the rat *Prl7b1* promoter. The regulatory region upstream of the *Prl7b1* locus is highly accessible in the invasive trophoblast cell cluster in contrast to other clusters. Each colored box indicates snATAC-seq profile of each cell cluster with a corresponding color. Panels B-D were generated from a re-analysis of previously published data (Scott et al., 2022; Vu et al., 2023). (E) Relative expression of *Prl7b1* transcript in various tissues determined by RT-qPCR. Data are shown as mean±s.e.m., *n*=5. ****P<0.0001 (one-way ANOVA).

The rat placentation site is arranged into three well-defined compartments: labyrinth zone, junctional zone and uterine–placental interface (UPI) (Shukla and Soares, 2022; Fig. 1A). The labyrinth zone is located at the placental–fetal interface adjacent to the junctional zone, which borders the uterine parenchyma. As gestation progresses, invasive trophoblast cells exit the junctional zone and infiltrate the uterine parenchyma, establishing a structure we define as the UPI, which has also been called the metrial gland.

### *Prl7b1* expression in the rat placentation site

**Fig. 2.**
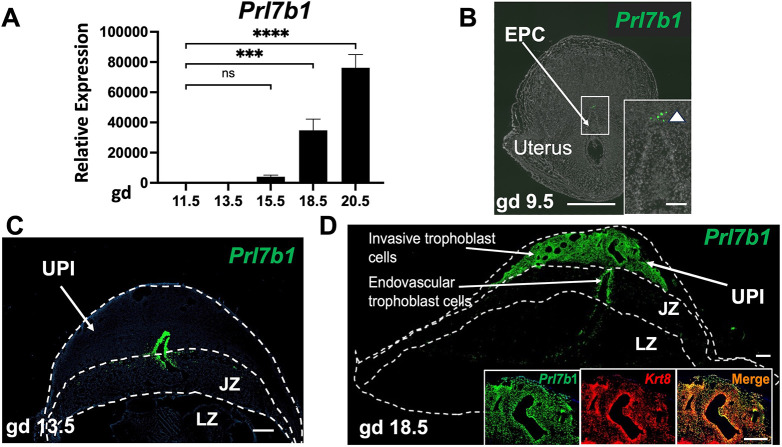
**Distribution of *Prl7b1*-expressing invasive trophoblast cells within the rat UPI.** (A) Relative expression of *Prl7b1* transcripts within the UPI during gestation as measured by RT-qPCR. Data are shown as mean±s.e.m., *n*=6 (3-6 pregnancies). ***P<0.001, ****P<0.0001 (ordinary one-way ANOVA with Dunnetts's multiple comparisons test). ns, not significant. (B) *In situ* hybridization localization of invasive trophoblast cell-specific *Prl7b1* transcripts within the gd 9.5 placentation site. (C) *In situ* hybridization localization of invasive trophoblast cell-specific *Prl7b1* transcripts within the gd 13.5 placentation site. (D) *In situ* hybridization localization of invasive trophoblast cell-specific *Prl7b1* transcripts within the gd 18.5 placentation site. Insets show co-localization of *Prl7b1* and *Krt8* transcripts within the UPI. EPC, ectoplacental cone; JZ, junctional zone; LZ, labyrinth zone. Scale bars: 1000 μm (B, main panel); 200 μm (B, inset); 500 μm (C;D, main panel); 100 μm (D, insets).

We recently performed single-cell RNA sequencing (scRNA-seq) and single nucleus assay for transposase-accessible chromatin using sequencing (snATAC-seq) for the rat UPI from gestation day (gd) 15.5 and 19.5 (Scott et al., 2022; Vu et al., 2023). *Prl7b1* was abundantly and specifically expressed within invasive trophoblast cells (Fig. 1B). At gd 19.5, 99.7% of invasive trophoblast cells located within the UPI express *Prl7b1* (Scott et al., 2022). snATAC-seq revealed that the regulatory region of *Prl7b1* is accessible in the invasive trophoblast cell cluster (Fig. 1C,D). Among several potential host genes for Cre recombinase, we selected the *Prl7b1* gene for further analysis. Reverse transcription-quantitative polymerase chain reaction (RT-qPCR) measurements demonstrated more abundant expression of *Prl7b1* transcripts in the UPI compared with any other tissue investigated (Fig. 1E). RT-qPCR quantification of UPI *Prl7b1* transcripts revealed progressive increases in expression as gestation proceeded (Fig. 2A). We confirmed the specificity of *Prl7b1* to invasive trophoblast cell lineage-specific expression by *in situ* hybridization of the UPI (Fig. 2B-D). *Prl7b1* transcripts were specifically localized to both endovascular and interstitial invasive trophoblast cells situated in the UPI.

### Effect of *Prl7b1* disruption on fertility and pregnancy outcomes

**Fig. 3.**
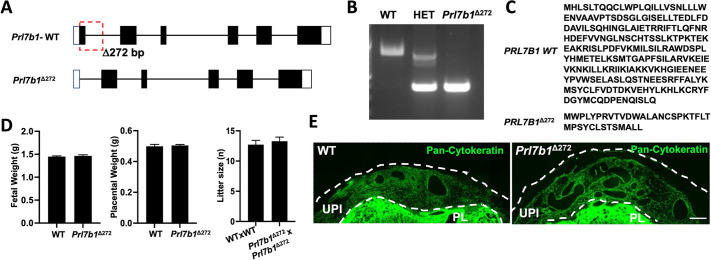
**Generation of the *Prl7b1* null rat and effects on pregnancy outcomes.** (A) Schematic of the organization of the *Prl7b1* gene, including the site for CRISPR/Cas9-mediated deletion (Δ272 bp). (B) Genotyping of wild-type and *Prl7b1* mutant alleles. Genomic DNA was isolated, PCR performed, and resolution of DNA fragments determined by agarose electrophoresis. (C) PRL7B1 wild-type and mutant amino acid sequences. (D) Placental and fetal weights of wild-type and *Prl7b1* null fetuses at gd 18.5 generated from *Prl7b1*^Δ272^ heterozygote mating and litter size generated from wild type×wild type versus homozygous *Prl7b1*^Δ272^×*Prl7b1*^Δ272^ mating. (E) Histological structure of wild type versus *Prl7b1*^Δ272^ placentation sites. Pan-cytokeratin staining of gd 18.5 placentation sites is shown within the uterus proximal to the placenta at gd 18.5. Pan-cytokeratin positive cells within the uterus proximal to the placenta identifies invasive trophoblast cells. HET, heterozygous; PL, placenta; WT, wild type. Scale bar: 500 μm.

A global *Prl7b1* deficient rat model was generated by CRISPR/Cas9 genome editing. A 272 bp deletion was generated that removed all of exon 1 and part of intron 1 (Fig. 3A-C). *Prl7b1*^Δ272^ heterozygote intercrosses generated litters of expected size and Mendelian ratio (Table S1). At birth, pups carrying *Prl7b1*^Δ272^ alleles (heterozygotes and homozygous nulls) were indistinguishable from wild-type littermates. Homozygous *Prl7b1*^Δ272^ rats were fertile and displayed no obvious phenotypic abnormalities. Consequently, some phenotypic comparisons were carried out on wild-type intercrosses versus homozygous *Prl7b1*^Δ272^ intercrosses. Phenotypic assessments were made at gd 13.5 and 18.5 (Fig. 3D). Litter size did not differ between wild-type and null pregnancies (Fig. 3D). Additionally, the organization of gd 13.5 placentation sites was not significantly affected by PRL7B1 deficiency (Fig. 3E). Junctional zone and labyrinth zone compartments were well-defined in both genotypes, as was the extent of intrauterine trophoblast invasion at gd 18.5 (Fig. 3E). Postnatal litter size and developmental outcomes were similar among wild-type intercross breeding and PRL7B1-deficient intercross breeding. Thus, PRL7B1 deficiency does not adversely affect fertility or pregnancy outcomes.

### Generation of *Prl7b1-iCre* knock-in

**Fig. 4.**
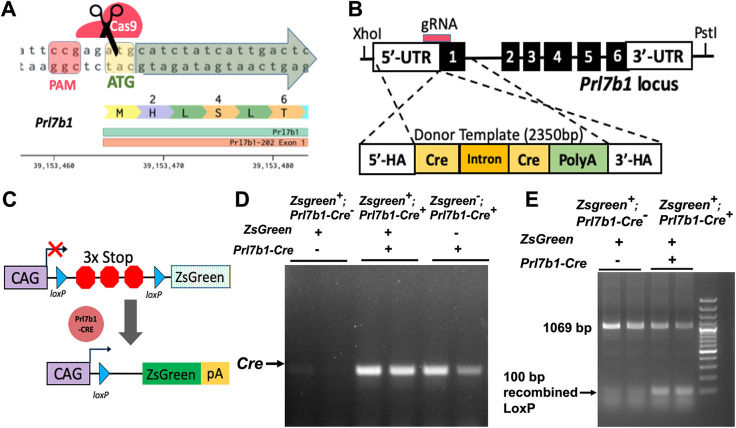
**Generation of the *Prl7b1-iCre* knock-in rat driver rat strain.** (A) Schematic of CRISPR/Cas9-mediated insertion of *iCre* within the *Prl7b1* locus. (B) Schematic of exon 1 of the wild-type *Prl7b1* locus. The CRISPR crRNA/tracrRNA target site is located close to the *Prl7b1* ATG start codon. (C) The reporter rat consists of a ubiquitously active CAG promoter (CAG), a LoxP-3x Stop-LoxP cassette (LSL) and ZsGreen (Bryda et al., 2019). The ZsGreen protein is expressed only where *Prl7b1-iCre* is expressed. (D) Cre transcript expression detected by RT-PCR in gd 18.5 UPI tissue isolated from *CAG-ZsGreen*×*Prl7b1-iCre* mating. (E) Genotyping of gd 18.5 UPI tissue isolated from *CAG-ZsGreen×Prl7b1-iCre* mating showing Cre-mediated loxP excision. HA, homology arm; pA, polyA; PAM, protospacer adjacent motif.

We used the CRISPR/Cas9 genome-editing technique to generate *Prl7b1-iCre* knock-in rats. We inserted the codon improved Cre (iCre) recombinase coding sequence immediately after the start codon in exon 1 of the *Prl7b1* locus, to generate a rat knock-in in which Cre expression faithfully recapitulates the endogenous spatial and temporal *Prl7b1* expression (Fig. 4A,B). Our *Prl7b1-iCre* rat strain was designed so that the endogenous *Prl7b1* regulatory elements drive expression of Cre recombinase. Founder rats possessing the appropriate insertion of *iCre* into the *Prl7b1* gene were backcrossed to wild-type rats to demonstrate successful germline transmission. Homozygous and heterozygous *Prl7b1-iCre* knock-in rats exhibited no apparent abnormalities, such as embryonic development and growth or iCre toxicity. Additionally, we did not observe any pregnancy and fertility phenotypes in *Prl7b1*-Cre homozygotes (Fig. S1).

### Characterization of the *Prl7b1-iCre* driver rat strain

**Fig. 5.**
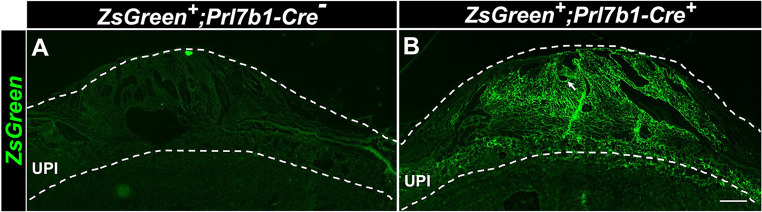
**Characterization of Cre activity at the histological level in the UPI of the *Prl7b1-Cre* transgenic rat model.** Cre recombinase-mediated expression of the ZsGreen reporter gene in *Prl7b1*-expressing invasive trophoblast cells in the UPI at gd 18.5. (A) ZsGreen fluorescence is not observed in *Zsgreen+;Prl7b1-iCre^−^* owing to the absence of Cre activity in the ZsGreen UPI. (B) ZsGreen fluorescence is specifically localized to the UPI as a result of *Prl7b1-Cre* recombinase-mediated recombination of the ZsGreen reporter gene. Arrow indicates ZsGreen fluorescence in invasive endovascular trophoblast cells. Scale bar: 500 μm.

**Fig. 6.**
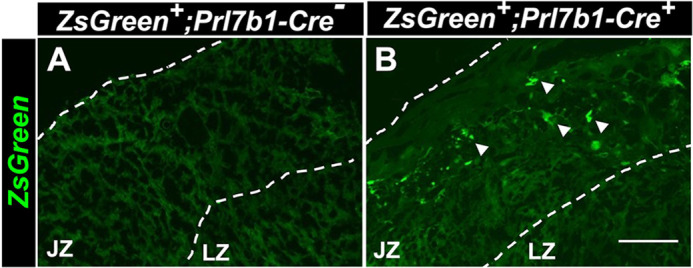
***Prl7b1*-driven Cre-mediated recombination during development in placental tissue.** (A) ZsGreen fluorescence is not observed owing to the absence of Cre activity in ZsGreen placenta. (B) Punctate ZsGreen fluorescence is observed in the junctional zone (arrowheads). Scale bar: 500 μm.

To test the efficiency and specificity of Cre recombinase activity in the *Prl7b1-iCre* rat, we mated heterozygous male *Prl7b1-iCre* rats with the Cre-dependent Tg(CAGloxP-STOP-loxP-ZsGreen) reporter line (Bryda et al., 2019). The reporter rat strain possesses a ZsGreen gene downstream of a floxed STOP cassette. When bred to a strain expressing Cre recombinase under the control of various tissue-specific promoters, loxP site-specific excision of the STOP cassette occurs resulting in expression of the ZsGreen gene driven by the ubiquitously and constitutively active chicken β-actin promoter coupled with the cytomegalovirus early enhancer (Fig. 4C). We initially confirmed the successful functioning of *Prl7b1-iCre* system by assessing expression of the *iCre* cDNA using RT-qPCR measurements of UPI tissues (Fig. 4D). We confirmed the Cre recombinase-mediated recombination of loxP and site-specific excision of STOP codons in UPI tissues by PCR (Fig. 4E). To examine tissue specificity of the *Prl7b1-iCre* rat line, we isolated UPI tissues and placentas from gd 18.5 pregnant rats and imaged tissue for ZsGreen fluorescence. We observed green fluorescence of ZsGreen throughout the UPI (Fig. 5A,B). The only *Prl7b1-iCre* positive cells capable of activating ZsGreen fluorescence within the UPI are invasive trophoblast cells. Within the placenta, ZsGreen-positive cells were observed in endovascular trophoblast cells lining central placental arteries and a small subset of cells situated within the junctional zone (Fig. 6A,B). The latter may be at least part of the invasive trophoblast progenitor cell population.

## DISCUSSION

Invasive trophoblast cells transform the uterus facilitating blood flow to the placenta and fetus (Red-Horse et al., 2004; Velicky et al., 2016). Disruption of trophoblast cell invasion and remodeling of uterine vasculature have been associated with obstetrical complications, including early pregnancy loss, pre-eclampsia, intrauterine growth restriction, and preterm birth (Kaufmann et al., 2003; Brosens et al., 2011). Thus, there is merit in understanding the regulatory mechanisms controlling the differentiation and function of the invasive trophoblast cell lineage. In this study, we established a rat model specifically expressing Cre recombinase in invasive trophoblast cells. Cre recombinase was incorporated into the *Prl7b1* locus using CRISPR/Cas9 genome editing and specifically activated in invasive trophoblast cells situated within the UPI.

The *Prl7b1* locus was selected as a host for *Cre* recombinase for two important reasons: (1) *Prl7bl* is expressed in an invasive trophoblast-specific pattern; and (2) disruption of the *Prl7b1* does not undermine placentation or pregnancy. PRL7B1 is a member of the expanded prolactin family and includes orthologs in rat and mouse, but not human (Soares, 2004; Soares et al., 2007). Both endovascular and interstitial invasive trophoblast cells situated within the UPI express *Prl7b1* (Wiemers et al., 2003; Scott et al., 2022; Vu et al., 2023). The absence of a detectable pregnancy-related phenotype in the *Prl7b1* mutant rat is consistent with experimentation with the *Prl7b1* mutant mouse (Bu et al., 2016). In the mouse, *Tpbpa* and *Prdm1* genes have been used to target Cre recombinase to trophoblast cells, including invasive trophoblast cells (Hu and Cross, 2011; Mould et al., 2012); however, neither locus directs expression exclusively to the invasive trophoblast cell lineage in the mouse or rat (Ain et al., 2003; Scott et al., 2022). The rat *Prl7b1* locus is optimal for directing mutations specifically to the invasive trophoblast cell lineage.

Effective tools for dissecting regulatory pathways controlling invasive trophoblast cells are limited. There is a plethora of experimental work performed *in vitro* with the goal of elucidating the mechanisms underlying invasive trophoblast cell differentiation and function. Much of this work is of limited value owing to inherent problems in using transformed or immortalized cell culture systems or because of the artificial nature of all *in vitro* analyses (Lee et al., 2016; Soares et al., 2018). Trophoblast stem cell models have greatly advanced the field and are best for generating hypotheses governing molecular mechanisms that can be tested *in vivo* (Tanaka et al., 1998; Asanoma et al., 2011; Okae et al., 2018). Conservation at structural and molecular levels within the human and rat UPI are evident (Pijnenborg and Vercruysse, 2010; Soares et al., 2012; Scott et al., 2022; Shukla and Soares, 2022; Vu et al., 2023). Global gene disruption in the rat has proven effective in gaining insights regarding the biology of some of the genes involved in regulating the invasive trophoblast cell lineage (Chakraborty et al., 2016; Muto et al., 2021; Varberg et al., 2021; Kozai et al., 2023; Kuna et al., 2023); however, it is apparent that genes potentially involved in controlling the biology of invasive trophoblast cells are also used in earlier phases of trophoblast cell development or in other aspects of embryonic or extra-embryonic development (Scott et al., 2022; Vu et al., 2023). The *Prl7b1-Cre* rat model provides a valuable tool for *in vivo* testing of hypotheses proposed to explain mechanisms controlling the biology of the invasive trophoblast cell lineage.

## MATERIALS AND METHODS

### Animals and tissue collection

Holtzman Sprague–Dawley rats were purchased from Envigo and maintained under specific pathogen-free conditions in an Association for Assessment and Accreditation of Laboratory Animal Care-accredited animal facility at the University of Kansas Medical Center. Rats were fed standard rat chow and water *ad libitum* and maintained in a 14-h light:10-h dark photoperiod (lights on at 06.00 h). Time-mated pregnancies were established by co-housing adult female rats (8-12 weeks of age) with adult male rats (>10 weeks of age). Detection of sperm or a seminal plug in the vagina was designated gd 0.5. Pseudopregnant females were generated by co-housing adult female rats (8-12 weeks of age) with adult vasectomized male rats (>10 weeks of age). At the time of euthanasia, litter sizes and the viability of conceptuses were recorded, and tissues used for histological analysis were frozen in dry ice-cooled heptane and stored at −80°C until processed, whereas tissues used for biochemical analyses were frozen in liquid nitrogen and stored at −80°C until processed (Ain et al., 2006; Chakraborty et al., 2011, 2016). All animal procedures were approved by the University of Kansas Medical Center Institutional Animal Care and Use Committee.

### Re-analysis of scRNA-seq and snATAC-seq

scRNA-seq data from rat UPI tissues (Scott et al., 2022) were downloaded from the Gene Expression Omnibus database (accession number GSE206086) and re-analyzed using Cell Ranger analysis pipelines (10x Genomics). Briefly, cellranger count was used to perform alignment, filtering, barcode counting, and UMI counting alignment. Output from multiple samples was combined using cellranger aggr (version 7.0.1). snATAC-seq data from uterine–placental tissues (Vu et al., 2023) was downloaded from the Gene Expression Omnibus database (accession number GSE227943) and re-analyzed using cellranger-atac analysis pipelines (10x Genomics). Briefly, cellranger-atac count was used for detection of accessible chromatin peaks, count matrix generation for peaks and transcription factors. Output from multiple samples was combined using cellranger-atac aggr (version 1.1.0). scRNA-seq and snATAC-seq data were visualized using Loupe Browser (10x Genomics).

### Transcript analysis

Total RNA was extracted from tissues using TRIzol reagent (AM9738, Thermo Fisher Scientific). cDNAs were synthesized from total RNA (1 μg) for each sample using SuperScript 2 reverse transcriptase (18064014, Thermo Fisher Scientific), diluted 1:10 with water, and subjected to RT-qPCR to estimate mRNA levels. RT-qPCR primer sequences are presented in Table S2. Real-time PCR amplification of cDNAs was carried out in a reaction mixture (20 μl) containing SYBR Green PCR Master Mix (4309155, Applied Biosystems) and primers (250 nM each). Amplification and fluorescence detection were carried out using the ABI QuantStudio PCR system (Applied Biosystems). The delta–delta Ct method was used for relative quantification of the amount of mRNA for each sample normalized to *18S* RNA.

### *In situ* hybridization

Distributions of transcripts for *Prl7b1* and *Krt8* were determined on 10-µm-thick cryosections (prepared using a Leica CM1850 cryostat, Leica Biosystems) of rat placentation sites at various gestation days. RNAScope Multiplex Fluorescent Reagent Kit version 2 (Advanced Cell Diagnostics) was used for *in situ* hybridization analysis. Probes were prepared to detect *Prl7b1* (NM_153738.1, 860181, target region: 28-900) and *Krt8* (NM_199370.1, 873041-C2, target region: 134-1,472). Fluorescence images were captured on a Nikon 80i upright microscope with a Photometrics CoolSNAP-ES monochrome camera (Roper Scientific Inc.). Images were processed using Fiji software.

### Generation of global *Prl7b1* knockout and *Prl7b1-iCre* knock-in rat models

The rat *Prl7b1* (ENSRNOG00000016742, NM_153738) gene is situated on chromosome 17 (Chr17: 39,153,434-39,161,643) among members of the prolactin (PRL) family. Mutations at the *Prl7b1* locus were generated using genome editing as previously described by our laboratory (Iqbal et al., 2007, 2009, 2011, 2021a,b; Kozai et al., 2021, 2023; Muto et al., 2021; Varberg et al., 2021; Kuna et al., 2023) with some modifications. In brief, 4- to 5-week-old donor rats were intraperitoneally injected with 30 units of equine chorionic gonadotropin (G4877, Sigma-Aldrich), followed by an intraperitoneal injection of 30 units of human chorionic gonadotropin (C1063, Sigma-Aldrich) ∼46 h later, and immediately mated with adult male rats. Zygotes were flushed from oviducts the next morning (gd 0.5) and maintained in M2 medium (MR-015-D, EMD Millipore) supplemented with bovine serum albumin (A9647, Sigma-Aldrich) at 37°C in 5% CO_2_ for 2 h. A CRISPR RNA (crRNA) sequence was designed to target exon 1 (AGTCAATGATAGATGCATCTCGG) and was located near the translation start codon (ATG) for the rat *Prl7b1* gene (NM_153738). crRNAs were annealed with tracrRNA in equimolar concentrations to generate crRNA:tracrRNA duplexes (guide RNA). The ribonucleoprotein (RNP) complex consisting of the Cas9 protein and a synthesized crRNA/tracrRNA along with a double-stranded deoxyribonucleic acid (dsDNA) donor template were microinjected into embryonic day (E) 0.5 rat zygotes at a concentration of 25 ng/μl in Tris-EDTA buffer (pH 7.4). The 2350 bp donor template consisted of an iCre cassette with a Kozak sequence and an intron (1100 bp) followed by a heterologous poly A signal sequence (200 bp) (Wu et al., 2008) and two 500 bp (1000 bp) homology arms (Fig. 4A,B). We incorporated Kozak and synthetic intron elements into the iCre transgene to increase transgene expression. The donor template DNA was excised from the plasmid backbone prior to co-microinjection with the RNP. Genome-editing reagents were obtained from Integrated DNA Technologies. Microinjections were performed using a Leica DMi6000 inverted microscope and an Eppendorf microinjection system (Eppendorf FemtoJet Microinjector 5247). Manipulated zygotes were transferred to oviducts of pseudopregnant rats (20-30 zygotes per rat). Offspring were screened for deletions and insertions of *iCre* sequence within the *Prl7b1* gene. Insertion boundaries were verified by Sanger DNA sequencing. PCR primers used for genotyping of the genetically altered rats are listed in Table S2. Germline transmission of the mutated gene was determined in F1 rats by backcrossing F0 founder rats with wild-type rats. Detection of a mutation in F1 rats identical to the mutation present in the parent F0 rat was considered successful germline transmission. *Prl7b1* global knockout and *Prl7b1-Cre* knock-in models will be available through the Rat Resource & Research Center (University of Missouri, Columbia, MO, USA; www.rrrc.us).

### Histological and immunohistochemical analyses

Immunohistochemical analyses were performed on 10-μm-thick frozen tissue sections using indirect immunofluorescence. Primary antibodies to vimentin (1:1000; V6630, Sigma-Aldrich) and pan-cytokeratin (1:1000; F3418, Sigma-Aldrich) were used in the analyses. Goat anti-mouse IgG conjugated with Alexa 488 (1:1000; A11029, Thermo Fisher Scientific) and goat anti-mouse IgG conjugated with Alexa 568 (1:400; A11031, Thermo Fisher Scientific) were used to detect primary antibodies. Fluoromount-G™, with 4′6-diamidino-2-phenylindole (DAPI; 00-4959-52, Thermo Fisher Scientific), was used to visualize nuclei and as mounting medium. Processed tissue sections were examined and images were captured with a Nikon Eclipse 80i upright microscope equipped with a charge-coupled device camera (Nikon DS-Fi3).

### Statistical analysis

Unpaired, one-tailed Student's *t*-test and ordinary one-way ANOVA followed by Dunnetts's multiple comparisons test were performed, where appropriate, to evaluate the significance of the experimental manipulations. Results were determined statistically significant when *P*<0.05.

## Supplementary Material

10.1242/develop.202239_sup1 Supplementary information
