# Supplementary material for: Conditionally mutant animal model for investigating the invasive trophoblast cell lineage
Source: Development. 2024 Jan 15;151(2):dev202239. doi: 10.1242/dev.202239 (PMC10820817; doi:10.1242/dev.202239)
Supplement: Supplementary information [file develop-151-202239-s1.pdf]

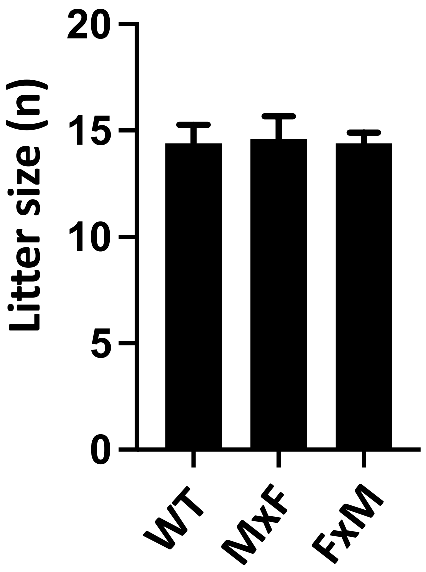

Fig. S1. *Prl7b1-iCre* insertion does not affect litter size. Breeding homozygous *Prl7b1-iCre* males and females in different combinations (Male x Female; **M x F**, Female x Male, **F x M**) resulted in a comparable number of offspring as observed to wild-type rats. (n=5 in each group).

Table S1. Mendelian ratio distribution of WT, heterozygous and homozygous progenies.

Cross: ***Prl7b1*** $^{\Delta 272}$ **X** ***Prl7b1*** $^{\Delta 272}$	**Pups Genotype**		
	Prl7b1 $^{+/+}$ (Wild Type)	***Prl7b1*** $^{+/\Delta 272}$ (Heterozygotes)	***Prl7b1*** $^{\Delta 272/\Delta 272}$ (Null)
Live pups	41	74	43
Proportion	25.9	46.8	27.2
Expected proportion	25	50	25

Table S2. Oligos used for genotyping

Gene	Forward	Reverse
Prl7b1	CCGTCATACTGTCTCAGCACATC	AGCTGTTGAGACCATTGACAACAAA
***Prl7b1-Cre* Genotyping primers**	AATGCTTCTGTCCGTTTGCC	CATTGCTGTCACTTGGTCGT

Development • Supplementary information
